# The Potential of Blockchain Technology in Dental Healthcare: A Literature Review

**DOI:** 10.3390/s23063277

**Published:** 2023-03-20

**Authors:** Takua Mokhamed, Manar Abu Talib, Mohammad Adel Moufti, Sohail Abbas, Faheem Khan

**Affiliations:** 1 Department of Computer Science, College of Computing and Informatics, University of Sharjah, Sharjah 27272, United Arab Emirates; 2 Department of Preventive and Restorative Dentistry, College of Dental Medicine, University of Sharjah, Sharjah 27272, United Arab Emirates; 3 Artificial Intelligence Lab, Department of Computer Engineering, Gachon University, Seongnam 13557, Republic of Korea

**Keywords:** blockchain, smart contract, dentistry, electronic dental records

## Abstract

Blockchain technology in the healthcare industry has potential to enable enhanced privacy, increased security, and an interoperable data record. Blockchain technology is being implemented in dental care systems to store and share medical information, improve insurance claims, and provide innovative dental data ledgers. Because the healthcare sector is a large and ever-growing industry, the use of blockchain technology would have many benefits. To improve dental care delivery, researchers advocate using blockchain technology and smart contracts due to their numerous advantages. In this research, we concentrate on blockchain-based dental care systems. In particular, we examine the current research literature, pinpoint issues with existing dental care systems, and consider how blockchain technology may be used to address these issues. Finally, the limitations of the proposed blockchain-based dental care systems are discussed which may be regarded as open issues.

## 1. Introduction

Over the past several years, blockchain has been an interesting study topic and many different industries have made use of its advantages [1]. In the healthcare sector including dental care, the use of blockchain technology can bring many benefits such as enhanced security, privacy, confidentiality, and decentralization [2]. Currently, the dental industry faces challenges related to data interoperability, security, privacy, and integrity [3]; implementing blockchain technology could offer a solution to these concerns. Its inherent features (e.g., encryption, hashed linkage model, consensus, and smart contract) provide a secure, tamper-proof platform for the storage of medical and dental records.

On a peer-to-peer network, a decentralized database known as the blockchain keeps track of transactions in an organized and hashed list of blocks [4]. This list of blocks is called Ledger. In a decentralized network, each node that creates transactions will employ a consensus process to maintain a consistent picture of the ledger. Instead of depending on a central, questionably trustworthy entity, its nodes can create confidence with one another using mathematical algorithms and cryptography. The ledger enables accountability and tracking of tamper-proof records. Users can choose to preserve their pseudo-anonymity while still being able to identify themselves if necessary [5]. Smart contracts, which are self-executing software programs, may be used on the blockchain network to improve functionality and robustness in a variety of applications [6]. Typically, they are utilized to automate and define the terms of an agreement between the participants of the blockchain network. Despite the many advantages of the blockchain, its adoption in the field of dentistry remains limited.

As a result, to advance the use of blockchain in dental research, we conducted this systematic literature review to examine the existing research articles that discuss data management technologies in dentistry and identify existing challenges. Then we investigated the potential of utilizing blockchain technology to solve these challenges. We collected 15 research papers on dentistry systems published during the last fourteen years (from 2009 to 2022) and 13 on blockchain-based dentistry systems published during the last four years (from 2019 to 2023) by renowned scientific publishers, such as IEEE, Elsevier, Hindawi, Springer, IGI-Global, and PubMed Central. To be as thorough as possible, we included both peer-reviewed and non-peer-reviewed papers, since there is not much research in the field of blockchain in dentistry. The key contributions of this study were identified in providing an analysis of research findings about existing data management technology in dentistry and identify the challenges. Further, we studied the proposed blockchain-based dental care solutions as potential for solving these challenges. Additionally, a detailed comparison of proposed blockchain-based solutions architectures was provided in terms of the location and blockchain framework. Finally, the challenges of blockchain-based dental care solutions are explored as open issues.

**Figure 1:**
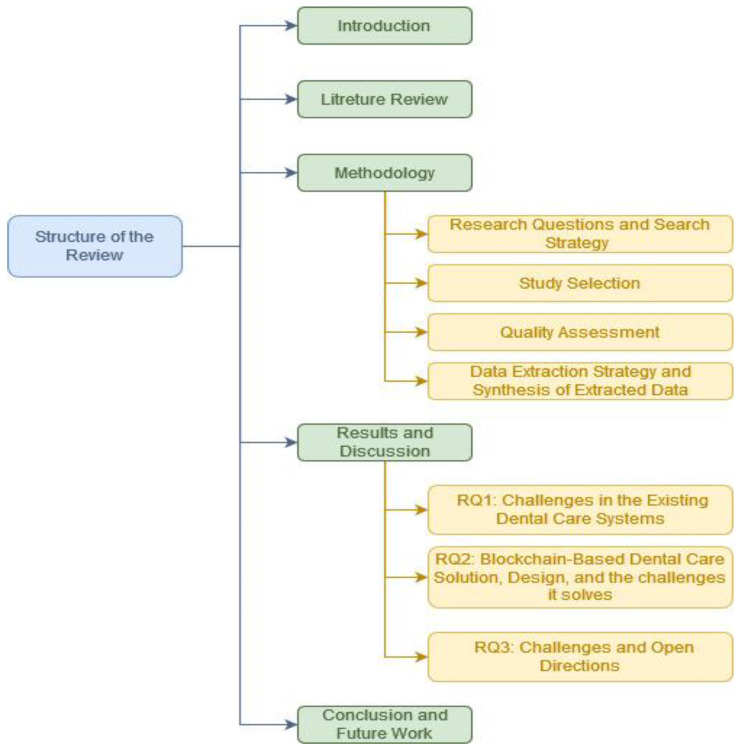
Structure of the Review.

The organization of this paper is showed in Figure 1. The related work and our contribution are outlined in Section 2. The methodology process for conducting this review study is provided in Section 3. A discussion of the results and findings of the selected articles is provided in Section 4. Finally, we sum up our research with a conclusion in Section 5.

## 2. Literature Review

The use of blockchain technology in healthcare is gaining popularity. Since the invention of this technology, there have been a rising number of review papers published in recent years. For example, several publications systematically reviewed the developed blockchain-based healthcare applications, as well as their shortcomings and difficulties, current efforts to solve these issues, and potential areas for development [7,8,9,10,11]. Nevertheless, various publications also surveyed the potential or implemented integration of blockchain, Internet of Dental Things (IoT), especially health IoT [12,13,14], and AI [15,16] for securing the storage and exchange of electronic health data. Moreover, the use of the blockchain has been studied in many subfields of healthcare. For instance, some researchers [17,18] have examined the existing implementations that adopt blockchain technology in the pharmaceutical supply chain industry. Further, Jadhav et al. [19] examined the research on how blockchain technology is altering the function of healthcare supply systems between different health providers. Alghazwi et al. [20] published on usage and implementation of blockchain technology in genomics.

The existing literature on the utilization of blockchain in the healthcare sector is summarized in Table 1. 

On the other hand, since blockchain is a relatively new technology, review papers concerning blockchain in dentistry are lacking. However, some published works have reviewed the use of new technologies in dentistry. Table 2 lists the year, primary contribution, and focus area of these review publications. Some articles provide a narrative overview of the key technologies of Dentistry 4.0 and its application in the dental field [27,28], such as Artificial Intelligence (AI), IoT, virtual reality, cloud computing, 5G technologies, dental digital scanners, robotics, and big data. Moreover, several research studies have examined the use of AI technology in the field of dentistry [29,30,31].

To our knowledge, there is no existing literature that specifically addresses blockchain-based solutions in the dental care industry, driving the motivation for this study. The selection process of articles was thorough and precise, taking into consideration the following aspects: (i) the challenges of the existing electronic dental record system (without blockchain) (ii) the proposed blockchain-based dental care systems including the blockchain platform utilized, methods of storing dental big data, and the challenges it solves (iii) the weaknesses/challenges of proposed solutions which will be considered as open issues for future study.

## 3. Methodology

According to Kitchenham and Charters [32], there are three key components of their technique: planning, conducting, and reporting. Each of these phases has distinct steps, with the creation of a review process being a vital aspect of the planning phase. The protocol (planning phase) should include the following items: (1) the definition of research objectives, (2) the creation of a search strategy, (3) the establishment of study selection criteria, (4) the development of quality evaluation guidelines, (5) the identification of data extraction methods, and (6) a description of how the gathered data will be consolidated. Further details on the recommended procedure can be found in the subsequent Section 3.1, Section 3.2, Section 3.3 and Section 3.4

### 3.1. Research Questions & Search Strategy

#### 3.1.1. Research Questions

The goal of this analysis is to evaluate the potential of blockchain technology to address difficulties in the current dental care system. With this purpose in mind, the following research questions (RQ) were formulated to guide the research:

1. **RQ1:** What are the problems and challenges of existing dental healthcare systems?
2. **RQ2:** Which challenges can be solved with blockchain technology? How would such proposed blockchain-based solutions be designed?
3. **RQ3:** What are open challenges and open issues?

#### 3.1.2. Search Strategy

The following criteria were used to conduct the search for articles on the use of blockchain in dentistry, as the availability of such articles is limited:

- Key search terms were derived based on the RQs.
- Additional terms were added to account for synonyms and variations in spelling.
- Boolean logic was employed using search operators (such as AND, OR, quotations, and parentheses) to increase the relevance of the search results.
- Due to the limited number of articles found, all papers, regardless of publication type (peer-reviewed journals, conferences, etc.), were included in the analysis.

Table 3 presents the search strings and criteria that yielded the most relevant results from several searches. 

We have searched the major electronic research repositories, including IEEE Xplore, ACM, Taylor and Francis, Hindawi, and Springer for the acquisition of pertinent papers for our Systematic Literature Review (SLR). However, certain works from IGI-Global, PubMed Central, the California Dental Association, Avicena Publishing, the Korean Academy of Oral and Maxillofacial Radiology, and Annual Reviews Inc. are also included in this study.

### 3.2. Study Selection

To ensure that only relevant and high-quality articles were included in the analysis, a filtering process was carried out. Both lead authors conducted this filtering independently to minimize any potential bias and resolve any discrepancies. The following steps were taken during the filtering process:

1. Duplicate and identical articles were removed.
2. To distinguish relevant articles and omit irrelevant works, certain inclusion, exclusion criteria, and quality assessment rules were used.

(a) The criteria for inclusion were as follows:
  - Articles dealing with dentistry and the use of blockchain technology for managing dental data and services.
  - Articles dealing with dentistry and the use of existing technology for managing dental data and services.
  - All papers, including those published in peer-reviewed journals and conference papers, were included.
(b) The criteria for exclusion were as follows:
  - Articles that pertained to blockchain but were not related to dental care.
  - Articles that pertained to dental care but were not related to blockchain or existing dental data and services management technologies.
(c) The quality assessment rules are explained in the Section 3.3.

### 3.3. Quality Assessment (QA)

Six quality assessment (QA) standards were established to evaluate the articles’ quality and identify their applicability to the research. Each quality assessment (QA) indicator was given a weight of 1, and the scores were as follows: “completely answered” = 1, “above average” = 0.75, “average” = 0.5, “below average” = 0.25, and “not answered” = 0. Each article received an overall quality score ranging from 0 to 6 by adding the scores of the various indicators. Only articles with an overall quality score of 3 or above were included. The following six quality indicators were used:

1. **QAR1:** Is the degree of detail used to describe the research objectives sufficient?
2. **QAR2:** Is the blockchain-based dental care system architecture explained with sufficient detail?
3. **QAR3:** Does the proposed system align well with the focus of this literature review?
4. **QAR4:** Does the suggested system solve the difficulties and challenges explored and reported?
5. **QAR5:** Are the strengths and limitations of the study analyzed explicitly?
6. **QAR6:** Does the study benefit the academic community or the business community?

### 3.4. Data Extraction Strategy and Synthesis of Extracted Data 

At this stage, a comprehensive assessment of the remaining included publications was conducted in order to collect the data required to respond to the RQs. A form was made to extract the pertinent information, and the extraction process was examined. The writers discussed any disagreements or misunderstandings that could have arisen between the extractor and checker. The extraction form had details on the paper’s title, publisher, year of publication, publication type (journal, conference, book chapter, etc.), and the author’s proposal for a blockchain-based dental care system. Each blockchain-based dental care system was analyzed, with the strengths and limitations of each system listed along with the blockchain platform and data storage type that were employed. It is worth mentioning that not all papers gathered could answer every RQ.

Several procedures were applied to combine the data acquired from the chosen publications and respond to the RQs. The narrative synthesis method was used to address each RQ. As part of the narrative synthesis, the data were represented graphically using pie charts, bar charts, and graphs.

## 4. Results and Discussion

**Figure 2:**
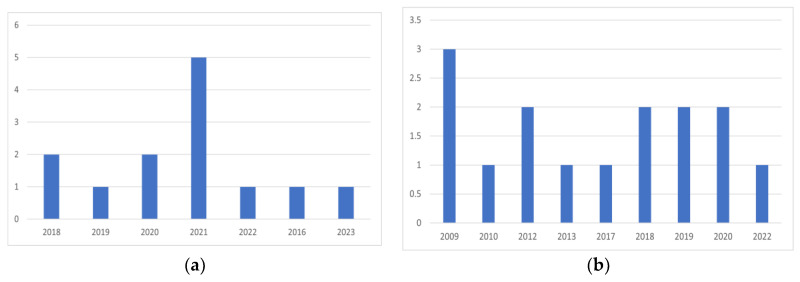
(**a**) Distribution of blockchain-dentistry-related publications over years; (**b**) Distribution of dentistry-related publications over years.

This section will explain the study’s findings. It also offers a summary of the academic articles selected to respond to the aforementioned RQs. The three parts that follow go into great detail on the findings of each RQ. Twenty-eight research publications were selected, 13 of which were blockchain-dentistry-related and 15 only dentistry-related. Table A1 of Appendix A contains a list of the selected research articles. The gathered blockchain-dentistry-related research publications were published between 2018 and 2023, whereas dentistry-related research publications were published between 2009 and 2022, as can be shown in Figure 2. As previously mentioned, a quality evaluation rule criterion was applied, and Table A2 displays the results for the selected publications.

### 4.1. The Problems and Challenges in the Existing Dental Healthcare Systems (RQ1)

Current-generation technologies are now being adopted more often in dental clinics in recent years. Adoption is encouraged for a number of reasons, such as expanding green practices, improved efficiency, digitalization of records, and sharing patient data with insurance companies, clinics, or other dental organizations [27]. Utilizing digital photography, radiography, and Electronic Dental Records (EDRs) are examples of green techniques that reduce waste creation [29]. Additionally, the employment of the aforementioned technologies such as EDR systems is seen as the tool for managing and storing the huge volume of patient data generated and accessed daily in the field of dentistry [33]. EDRs or Electronic Medical Records (EMR) comprise vital digital records and information on individuals such as their dental status, diagnoses, treatment plan, history, patient scheduling, report generation, and billing tasks management [29]. They also include personal and general medical information. Moreover, digital dental photographs and radiographs are also stored in EDR. As a result, ease of access to the patient’s dental history, readable and consistent writing, information system and other integrated bases, improved precision in using and analyzing data from imaging and laboratory equipment, and the updating of data are additional advantages of EDRs [3]. However, there are some clinics relying on paper-based recordkeeping approaches, as the computerized recordkeeping approach may have security, privacy, accessibility, and implementation complexity issues [33]. 

EDRs and information management system design and implementation inherit a number of difficulties, but one of the most significant is the dental record interoperability. Currently, systems at various clinics are fragmented and disconnected from each other [34]. At some clinics, the exchange of patients’ data between dental care providers is made through a manufacturer portal based on the local or global server storage, hence the dental company has access and control of the data [35,36]. This approach of data exchange is limited to homogeneous organizations that operate the same EDR software system, and this approach is not interoperable between various dental care organizations [37,38]. Therefore, each dental clinic has its own EDR system where patient records are maintained independently from other dental clinics’ databases. Consequently, patient dental and medical data could be scattered in different dental organizations, and this has negative effects including poor care coordination between dental care providers and insurance companies, and poor follow up of patient information across multiple dental care organizations. Alternatively, the data can be sent by email, which is not secure, or portable memory devices, which is inconvenient.

In response to these risks, attempts were made to develop digital dental care platforms for unified medical record through the centralization of dental records and the delivery of a completely integrated, digitalized clinical information system [39]. In this manner, interoperability and data sharing can be established. However, a system that combines and centralizes all patient EMR/EDR data still has several drawbacks. When EMRs/EDRs are kept in traditional database-centric architecture, these systems may struggle with data immutability and security. Even though healthcare and dental care providers and governmental organizations assert that EHRs/EDRs comply with the Health Insurance Portability and Accountability Act (HIPAA), some individuals worry about privacy concerns and data breaches when these systems are out of their control. Furthermore, the centralized storage architecture is thought to be a single point of failure with a high chance of privacy and data breaches with negative implications [40]. Another drawback is that the centralized systems create many issues related to access controls and consistency when different systems try to access or upload data simultaneously [41]. 

**Figure 3:**
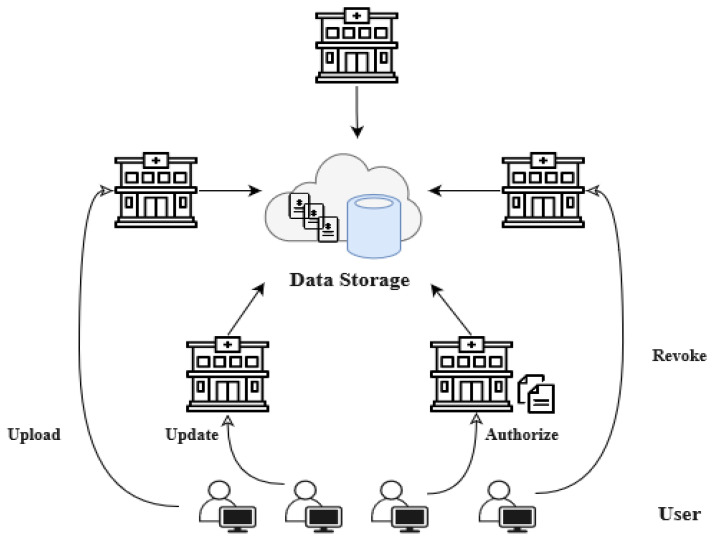
The general architecture for existing EDRs.

Figure 3 represents the general architecture for existing EDR systems where the data stored on the central or local storage. Clinics that have the same vendor software and access rights can retrieve from and upload the dental data into global storage [36,41,42]. However, in case of the local storage architecture, each clinic has its own storage which cannot be directly shared with other dental providers.

The summary of the most frequently occurring challenges in dental data storing and management systems are provided in Table 4.

### 4.2. The Challenges Blockchain Technology Can Solve and the Blockchain-Based Solution’s Design (RQ2)

This section aims to identify the challenges and difficulties that blockchain-based dental care solutions could potentially solve to address RQ1. Additionally, it presents the data location strategies and blockchain platforms used in the implementation of proposed blockchain-based EDR solutions.

**Figure 4:**
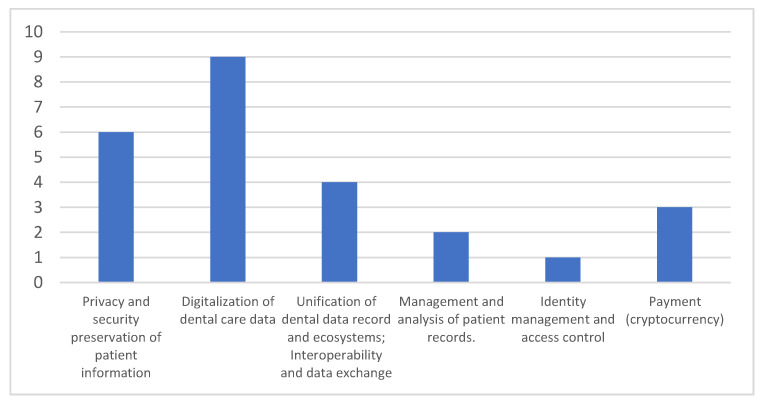
Problems solved by blockchain-based solutions in Dentistry.

As mentioned in the prior section, the existing dental care systems encounter a number of issues with data and service management, security, and integrity. However, blockchain technology’s built-in properties such as security, privacy, secrecy, decentralization, and automation can provide the dental care industry with several solutions to these issues. Table 5 demonstrates the difficulties overcome by the use of blockchain in the development of EDR systems, and the blockchain’s feature or characteristics which can resolve this difficulty. According to Figure 4, many studies propose a blockchain-based dentistry solution to address the issues, and each paper may address multiple issues.

In order to build a decentralized EDR system, the main targets in the implementation of most research was defined based on the specifications of an EDR system and the properties of blockchain [48,49,50,51,52,53,54,55,56]; these are outlined in Table 6 [23].

**Figure 5:**
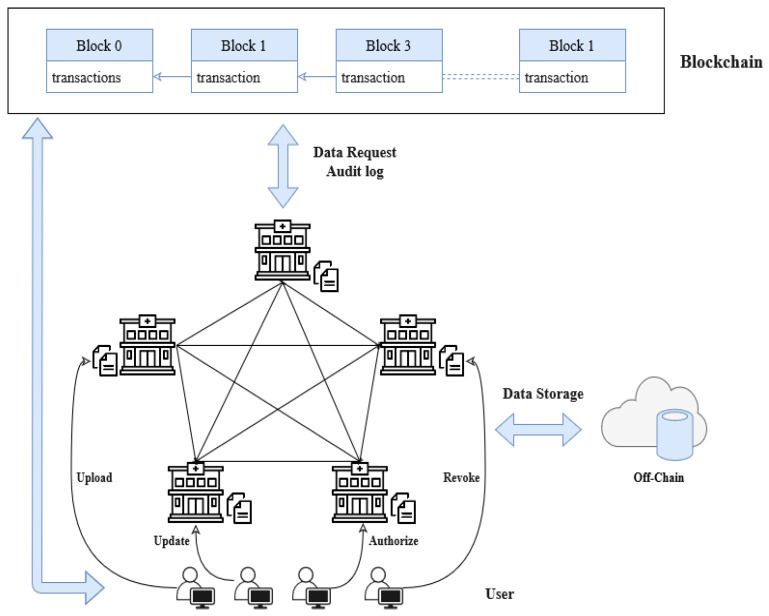
Blockchain-based EDR system: A top-level view.

To satisfy the above goals, the proposed blockchain-based EDR systems include the following main aspects:

- **Data storage:** For the storage of a variety of private dental care data, blockchain serves as a trustworthy ledger database [5]. When secure storage is established, data privacy should be guaranteed [9]. In reality, the volume of data (dental images) related to dental treatment is typically large [3]. Due to the set and constrained block size, storing these data directly in the blockchain network would result in increased computational and storage costs [8]. Consequently, handling massive data storage issues without negatively affecting the functionality of the blockchain network, could be solved by the following architecture.
  *Off-chain storage architecture*. In this architecture, blockchain is utilized for on-chain verification which only keeps certain metadata and pointers, such as the location of the matching raw data in the off-chain databases of the enormous quantities of encrypted original data [5,7,8]. The off-chain or cloud server storage will contain dental images and detailed dental records [3,5]. In this way, the integrity and privacy of private data are secured and the load of blockchain storage is reduced. The general architecture proposed by several researchers is illustrated in Figure 5 [48,49,50,51,52,55,57,58,59].
- **Data sharing:** Service providers using most current medical systems maintain primary data management. With the safe sharing of historical patient data (ledger) using blockchain, there is a movement to handle control of medical data and patients’ information between many dental care providers [9,10,60]. The controlled handling aspect should be specified in the smart contract [2].
- **Data request audit log:** When conflicts occur, audit logs (on-chain) can be used as evidence to hold requestors responsible for their interactions with EDRs [8]. The system will use a smart contract deployed on the blockchain network to maintain a trail for auditability and any action taken, including requests, will be noted on the blockchain ledger [6].
- **Identity manager:** The system must ensure that each user’s identification is authentic [4]. To maintain system security and prevent malicious attacks, only authorized users are permitted to submit the necessary requests [1]. It is preferable to utilize a private blockchain platform for implementation [4,7].

Table 7 shows the comparison between proposed blockchain solutions in dentistry in terms of the implementation of data storage for big data volumes, blockchain platforms, and main contribution. There were three main approaches of data location storage utilized in the designs proposed by research papers: on-chain, off-chain, and Inter Planetary File System (IPFS). Moreover, the platforms most frequently utilized are Ethereum and Hyperledger fabric, as these platforms are open-source and the network can be customized to be private or public. Another notable feature is that Ethereum and Hyperledger Fabric are smart-contract-based platforms, thus they not only digitally simplify the processes of verifying, controlling, or carrying out an agreement, but also provide a wide range of functions for various blockchain applications.

### 4.3. Challenges and Open Issues (RQ3)

The blockchain’s beneficial enhancements to security, privacy, access control, and distributed data sharing come with open issues and challenges that the research community has to address in order to improve the network’s infrastructure and design.

The block-size constraint characteristic of blockchain presents a limitation and constraint, because most of the systems described in the research stored textual records in the ledger [48,49,50,51,52,53,54,55,56,57,58], and only theoretically implemented a system for storing dental images in the blockchain [55,56]. Real implementations with experiments are required to assist the reliability of the system. 

Other key problems are the scalability, interoperability, transactions throughput, and latency assessment experiment. All research publications merely suggested blockchain-based dental care systems without doing proper evaluation analyses on the proposed solutions. The scalability and transactional latency test are necessary because it would be difficult for the blockchain and all of the participating nodes to function if many nodes/peers (dental clinics) were added to the blockchain network and sent data/transactions that scaled to a larger size at the same time. Additionally, the input/output operations and smart contract restrictions may cause transaction latency to rise, which presents a potential area for future research. 

In addition, almost all study articles used a theoretical approach without actually implementing the system, which might lead to overly optimistic conclusions for the delay in communication measures. Future work should take this issue into account especially in use of blockchain [57,58,59,60,61] of vehicular network [62] and wireless network [63,64,65,66,67] through systematic literature review [67,68,69]. 

Last but not least, the lack of a sufficient blockchain foundation for healthcare is another key obstacle to application, making it challenging to scale blockchain systems in actual use [8]. Although combining blockchain-based patient health records with an existing health data system is a more practical plan, the system would need to be rebuilt, new personnel would need to be trained and hired, and directors could need persuading that blockchain technology is a worthwhile investment [29,34]. Health-related companies’ main responsibility in the short term is to support system improvement [27,35,37,38,40].

## 5. Conclusions

The introduction of this technology to the dental area of healthcare provision remains restricted, although efforts have been made to enhance the marketing of blockchain use in dentistry (cryptocurrency) and for general dental clinics. Nevertheless, blockchain technology has numerous uses and advantages in healthcare. Thus, we analyzed this technology for a specific area of dentistry practice with the primary objective of identifying the problems with the current dental healthcare data and service management systems and the potential of blockchain to address these problems. In order to facilitate future research on blockchain in the field of dentistry, we also highlighted the challenges faced by the blockchain-based dental care system.

Based on our systematic review using the Kitchenham methodology, we have found that blockchain technology has the potential to revolutionize dental healthcare by providing secure, decentralized, and transparent data storage and sharing. The majority of the studies we analyzed indicate that blockchain technology can enhance patient privacy and security, improve data management and sharing, automate service, reduce fraud and errors, and facilitate supply chain management. However, there are also challenges associated with the adoption of blockchain technology in dental healthcare, such as regulatory barriers, technical limitations, and the need for specialized expertise.

In conclusion, the use of blockchain technology in dental healthcare is a promising area of research that warrants further investigation. While there is still much work to be done to fully realize the potential benefits of blockchain technology in this field, the evidence suggests that it has the potential to transform the way we approach dental healthcare. Future research should focus on addressing the challenges associated with blockchain adoption in dental healthcare and exploring ways to optimize its use for maximum impact.

## Figures and Tables

**Table 1:** Healthcare-related works: a comparative analysis.

Ref.	Year	Contributions	Focus
[7,8,9,10,11]	2018–2019	Systematic reviews and Surveys on emerging blockchain-based healthcare technologies and related applications	Blockchain in Healthcare
[21,22]	2020	Survey on the privacy and security issues in healthcare and blockchain-based solution	Privacy and Security in Healthcare
[23,24,25]	2020, 2021, 2022	Survey potential applications and developed solutions of blockchain for the management and storing of Electronic Health Records (EHRs)	EHR and Blockchain
[12,13,14]	2019, 2020, 2022	Literature Review of the Blockchain and IoT technologies in the healthcare industry to improve information security management	Integrating Blockchain with Healthcare IoT
[26]	2020	Survey on the development and methods for implementing blockchain and IoT in healthcare associated with the pharmaceutical industry	Blockchain and IoT in Pharmaceutical Supply Chain Industry
[17,18]	2022	Systematic literature review on existing pharmaceutical supply chain studies that adopt blockchain technology	Blockchain in Pharmaceutical Supply Chain Industry
[19]	2022	Overview of the literature on how blockchain technology is changing the way healthcare supply chains operate	Blockchain in Healthcare Supply Chain
[20]	2022	Systematic literature review on the applications of using blockchain and approaches and combinations of technologies used in genomics	Blockchain in Genomics
[15,16]	2021, 2022	Systematic Review on blockchain and AI solutions for advancing new technological standards for the healthcare ecosystem connected to medical diagnostics and the exchange of electronic health data	Integration of Blockchain and AI in secure EHR sharing

**Table 2:** Dentistry-related works: a comparative analysis.

Ref.	Year	Contributions	Focus
[27]	2021	Review of fifteen important Dentistry 4.0 technologies to the dental field in response to the COVID-19 pandemic	Dentistry 4.0 Technologies
[29]	2019	Review and discusses the difficulties managing health data and look ahead to the possibilities of AI in dentistry research and oral healthcare	AI
[28]	2022	Reviews the innovative technologies’ potential to disrupt the dental industry	Dentistry 4.0 Technologies
[30]	2021	Review on Big Data, artificial intelligence, and computerized patient dental data	AI and Big Data
[31]	2021	A systematic review of the artificial intelligence technologies implemented in a range of dentistry specialties	AI
Our Paper	2023	A systematic review of dental care systems’ challenges and the potential for blockchain-based solutions to address them	Blockchain

**Table 3:** Search strings used for LR.

Keywords	Search Strings
Blockchain	“Blockchain” OR “Digital ledger” OR “Shared ledger” OR “Distributed ledger” OR “Smart contract”
Dentistry	“Dentistry” OR “Dental care” OR “Oral health” OR “Dental Records” OR “Dental electronic health record”

**Table 4:** Challenges related to existing EDR systems.

Ref.	Challenges
[33]	Digitalization of dental care records.
[34,43]	Incomplete and fragmented patient’s dental data keeping manner. Consistency of patient’s records. Interoperability; patients’ dental record sharing/exchange between heterogeneous system
[35,36,40,42,44,45]	Privacy and security preservation of patients’ personal, dental, medical records.
[37,38,46]	Management of dental care services related to the dental clinic.
[39,41,47]	Identity management and access control.

**Table 5:** Problems solved by blockchain-based solutions.

Ref.	Challenges Blockchain-Based Solution Solves	Blockchain Feature
[48,49]	Privacy and security preservation of patient information	Encryption process and hashing structure
[48,49,50,51,52,53,54,55,56]	Digitalization of dental/health care data	On-chain ledger and cryptographic technologies
[51,53,56,57]	Unification of dental data record and ecosystems; Interoperability, data exchange, sharing, and consistency.	Peer-to-Peer, decentralization, consensus
[52,58]	Management and analysis of patient record.	Detailed audit logs in the on-chain ledger
[56]	Identity management and access control	Smart contract and Blockchain Type (Public or Private)
[38,56,59]	Payment	Cryptocurrency and Native tokens

**Table 6:** Targeted blockchain-based EDR system properties.

Properties	Description
Privacy	The protection of personal information, with access restricted to only authorized parties.
Security	The assurance of the CIA’s three pillars of security: confidentiality, integrity, and availability: Confidentiality: Only authorized individuals are allowed to access the data. Integrity: The accuracy of data must be maintained during transit and protected from unauthorized alterations. Availability: Information and resources must be readily accessible to those who are entitled to it.
Auditability	The ability to monitor and record access to information, including who accessed it and when.
Accountability	Audits are conducted to determine if any misconduct has occurred by individuals or organizations
Authenticity	The verification of the identity of those requesting access to critical information.
Immutability	The unalterable nature of data due to the use of cryptographic techniques.
Interoperability	The seamless communication and data sharing capabilities between various network nodes.

**Table 7:** Comparative analysis of blockchain-based EDR systems’ architecture.

**Ref.**	**Data Location**	**Blockchain** **Platform**	**Main Contribution**
[58]	On-chain	Hyperledger	Prototype for a healthcare service application in dental clinics. Information is stored on the blockchain through transaction and asset calls. Transaction number is linked to the dentist and patient.
[48]	Off-chain	Ethereum	Smart contract system developed to protect patient privacy and digitize healthcare data. Smart contract was written using the Solidity programming language. Built on a proof of authority blockchain network.
[50]	On-chain	Ethereum	Smart contract system for creating treatment plans involving multiple partners. Uses five models proposed in smart contract system. Programmed using Solidity. Operates on a proof of authority algorithm.
[51]	On-chain	not mentioned	Elements for the integration of diverse ecosystems and a comprehensive dental data record. System design model to transform dental data into the DVI INTERPOL standard and save on a blockchain-based distributed ledger.
[52]	On-chain	not mentioned	Management of patient data, appointment scheduling, cashless payment transactions, and safe patient reports for expert dentists during and after the COVID-19 epidemic.
[38]	not mentioned	not mentioned	Discusses idea of self-managing system based on blockchain, cryptocurrencies, and encouraging customer behavior as new business model for dentistry sector.
[53]	not mentioned	not mentioned	Focuses on the possible benefits that blockchain technology might bring to oral and maxillofacial radiography. Explains these benefits, including data sharing and security.
[54]	Off-chain	not mentioned	Smart medical blockchain technology-based application system. Improve the security of propofol in the anesthesia for oral and maxillofacial surgery. Trustworthy transactions and facilitates the exchange and interaction of medical data.
[55]	IPFS	not mentioned	Integration IPFS and blockchain technology. Allows doctors to conserve network bandwidth on the blockchain by eliminating the requirement to store data directly on the chain.
[59]	On-chain	Ethereum	Utilizes blockchain and smart contracts on the Ethereum platform to enhance the global dental market and promote preventive care.
[49]	On-chain	Hyperledger	Patients’ data reports and files uploaded were converted into Blockchain.
[56]	IPFS	Ethereum	Decentralized solution using blockchain technology and smart contracts to address challenges in EDR systems. Provides standardization, security, and gives patients control over their information.
[57]	On-chain	not mentioned	Use of blockchain to address challenges in 3D printing for digital dentistry. Proposed framework enables secure data and resource sharing. Utilizes prosthetic rebuilding efforts, CAD/CAM features, and 3D printing capabilities through an integrated network.

## Data Availability

Not applicable.

## Appendix A

**Table A1:** Selected Blockchain-Dentistry-related and Dentistry-related Research Papers.

Ref.	Title (Blockchain-Dentistry-Related)	Type	Year	Ref.	Title (Dentistry-Related)	Type	Year
[58]	Prototype of blockchain in dental care service application based on hyperledger composer in hyperledger fabric framework	Conference	2018	[33]	Information systems in dentistry.	Journal	2012
[48]	Blockchain and smart contracts to improve dental healthcare for children in primary school	Conference	2021	[34]	Patient-Centric Authorization Framework for Sharing Electronic Health Records,	Conference	2009
[50]	Blockchain in healthcare: smart contracts to improve dental healthcare for children in mixed dentition period	Thesis	2021	[35]	A novel digital dentistry platform based on cloud manufacturing paradigm	Journal	2019
[51]	Towards a unified blockchain-based dental record ecosystem for disaster victims’ identification.	Conference	2019	[36]	Privacy Preserving and Security Management in Cloud-Based Electronic Health Records	Conference	2020
[52]	A decentralised blockchain dentistry during and after the COVID 19 crisis	N/A	2022	[37]	The virtual dental home: implications for policy and strategy.	Journal	2012
[38]	Creating a platform-based business model in dental industry	Book Chapter	2018	[38]	Creating a Platform Based Business Model in Dental Industry	Conference	2018
[53]	What can blockchain technology bring to oral and maxillofacial radiology?	Journal	2020	[39]	Access Control Model for Sharing Composite Electronic Health Records	Conference	2009
[54]	Application of propofol in oral and maxillofacial surgery anesthesia based on smart medical blockchain technology	Journal	2021	[40]	The ethics of social media in dental practice: ethical tools and professional responses	Journal	2013
[55]	Can the blockchain-enabled interplanetary file system (block-ipfs) be a solution for securely transferring imaging data for artificial intelligence research in oral and maxillofacial radiology?	Journal	2021	[41]	Disparities in Access to Oral Health Care	Journal	2020
[59]	Dentacoin: a blockchain-based concept for dental healthcare	Book Chapter	2021	[42]	A fog-based security model for electronic medical records in the cloud database	Journal	2019
[49]	Blockchain: a new data standard in oral and maxillofacial radiology?	Journal	2020	[43]	Population-Based Linkage of Big Data in Dental Research	Journal	2018
[56]	Dental records on the blockchain	Thesis	2016	[44]	Social Media and Dentistry: Part 8: Ethical, legal, and professional concerns with the use of internet sites by health care professionals	Journal	2017
[57]	Using blockchain technology for 3d printing in manufacturing of dental implants in digital dentistry	Conference	2023	[45]	Forensic Dentistry: Electronic Transmission of Computerized Records	Journal	2009
				[47]	Online dental information database for dental identification system	Conference	2010
				[46]	Allocation and scheduling of digital dentistry services in a dental cloud manufacturing system	Journal	2022

**Table A2:** QAR Scores.

Ref.	QAR1	QAR2	QAR3	QAR4	QAR5	QAR6	Total Points
[58]	1	1	1	1	1	1	6
[48]	1	1	1	0.75	0.75	1	5.5
[50]	1	0.75	0.75	1	0.5	1	5
[51]	1	0.75	0.75	0.5	0.5	1	4.25
[52]	1	0.75	1	1	1	1	5.75
[38]	1	0.75	1	1	0.25	1	5
[53]	1	1	0.5	0.5	0.25	1	4.25
[54]	1	1	0.5	1	0.5	1	5
[55]	1	1	1	0.25	0.75	1	5
[59]	1	0.75	0.75	0.25	0.75	1	4.6
[49]	0.75	0.5	0.5	0.25	0.25	1	3.25
[56]	1	0.75	0.5	0.5	0.75	1	4.5
[57]	1	0.75	0.75	1	0.5	1	5
[33]	1	1	1	1	1	1	6
[34]	1	0.5	0.5	0.75	0.75	1	3.5
[35]	1	0.25	0.75	1	0.5	1	3.5
[36]	1	0.75	0.75	0.5	0.5	1	4.25
[37]	1	0.75	1	1	1	1	5.75
[38]	1	0.75	1	1	0.25	1	5
[39]	1	1	0.5	0.5	0.25	1	4.25
[40]	1	1	0.5	1	0.5	1	5
[41]	1	1	1	0.25	0.75	1	5
[42]	1	0.75	0.75	0.25	0.75	1	4.6
[43]	0.75	0.5	0.5	0.25	0.25	1	3.25
[44]	1	0.75	0.5	0.5	0.75	1	4.5
[45]	0.75	0.5	0.5	0.25	0.25	1	3.25
[47]	1	1	1	0.75	0.75	1	5.5
[46]	1	0.75	0.5	0.5	0.75	1	4.5
